# Synchrotron-Based Pencil Beam Scanning Nozzle with an Integrated Mini-Ridge Filter: A Dosimetric Study to Optimize Treatment Delivery

**DOI:** 10.3390/cancers9120170

**Published:** 2017-12-13

**Authors:** Xianliang Wang, Yupeng Li, Xiaodong Zhang, Heng Li, Koichi Miyazaki, Rintaro Fujimoto, Hiroshi Akiyama, Michael T. Gillin, Falk Poenisch, Narayan Sahoo, David Grosshans, Brandon Gunn, Steven Jay Frank, Pei Wang, Jinyi Lang, Qing Hou, Xiaorong Ronald Zhu

**Affiliations:** 1 Department of Radiation Physics, The University of Texas MD Anderson Cancer Center, Houston, TX 77054, USA; wangliu8687@163.com (X.W.); yupli@mdanderson.org (Y.L.); xizhang@mdanderson.org (X.Z.); hengli@mdanderson.org (H.L.); mgillin@mdanderson.org (M.T.G.); fpoenisch@mdanderson.org (F.P.); nsahoo@mdanderson.org (N.S.); 2 Department of Radiation Oncology, Sichuan Cancer Hospital & Institute, Chengdu 610041, China; dengwangpei@163.com (P.W.); langjy610@163.com (J.L.); 3 Key Laboratory of Radiation Physics and Technology, Institute of Nuclear Science and Technology, Sichuan University, Chengdu 610064, China; qhou@scu.edu.cn; 4 Research & Development Group, Hitachi, Ltd., Hitachi-shi, Ibaraki-ken 3178511, Japan; koichi.miyazaki.kj@hitachi.com (K.M.); rintaro.fujimoto.ns@hitachi.com (R.F.); 5 Healthcare Business Unit, Particle Therapy Division, Hitachi, Ltd., Hitachi-shi, Ibaraki-ken 3178511, Japan; hiroshi.akiyama.qj@hitachi.com; 6 Department of Radiation Oncology, The University of Texas MD Anderson Cancer Center, Houston, TX 77030, USA; dgrossha@mdanderson.org (D.G.); gbgunn@mdanderson.org (B.G.); sjfrank@mdanderson.org (S.J.F.)

**Keywords:** proton therapy, ridge filter, pencil beam scanning

## Abstract

A mini-ridge filter is often used to widen the Bragg peak in the longitudinal direction at low energies but not high energies. To facilitate the clinical use of a mini-ridge filter, we performed a planning study for the feasibility of a mini-ridge filter as an integral part of the synchrotron nozzle (IMRF). Dose models with and without IMRF were commissioned in a commercial Treatment planning system (TPS). Dosimetric characteristics in a homogenous water phantom were compared between plans with and without IMRF for a fixed spread-out Bragg peak width of 4 cm with distal ranges varying from 8 to 30 g/cm^2^. Six clinical cases were then used to compare the plan quality between plans. The delivery efficiency was also compared between plans in both the phantom and the clinical cases. The Bragg peak width was increased by 0.18 cm at the lowest energy and by only about 0.04 cm at the highest energy. The IMRF increased the spot size (σ) by up to 0.1 cm at the lowest energy and by only 0.02 cm at the highest energy. For the phantom, the IMRF negligibly affected dose at high energies but increased the lateral penumbra by up to 0.12 cm and the distal penumbra by up to 0.06 cm at low energies. For the clinical cases, the IMRF slightly increased dose to the organs at risk. However, the beam delivery time was reduced from 18.5% to 47.1% for the lung, brain, scalp, and head and neck cases, and dose uniformities of target were improved up to 2.9% for these cases owing to the reduced minimum monitor unit effect. In conclusion, integrating a mini-ridge filter into a synchrotron nozzle is feasible for improving treatment efficiency without significantly sacrificing the plan quality.

## 1. Introduction

In recent years, the technology for proton therapy delivery has advanced dramatically. As new centers open, the number of patients receiving proton therapy has also increased. Two types of proton accelerators, synchrotron and cyclotron, are available in clinical applications [1]. An important distinction between synchrotrons and cyclotrons is the energy levels available from the accelerators. A large number of energy layers can be generated by a synchrotron, whereas only one energy level normally is produced by a cyclotron. An energy selection system, including an energy degrader, is normally used to produce various desired energies for a cyclotron system [1]. A cyclotron system requires additional considerations in radiation shielding surrounding the energy selection system. A synchrotron system does not need an energy selection system and thus does not need additional radiation shielding. A synchrotron produces sharper Bragg peaks at lower energies. For a shallow target, many energy layers are needed to uniformly cover the target. The energy switching time of a synchrotron is typically about 2 s [2], so the irradiation time of pencil beam scanning (PBS) is heavily dependent on the number of energy layers. Thus, reducing the number of energy layers would improve the beam delivery efficiency.

One strategy to reduce the number of energy layers for each beam is to use a thick range shifter so that the lowest-energy layers are not used. This strategy has several disadvantages; for example, the maximum range available for patient treatment is reduced, which may make it difficult to cover large target volumes. However, if a thinner range shifter is used, then the number of energy layers may not be reduced. In addition, the use of multiple range shifters increases efforts on commissioning, treatment planning system (TPS) beam modeling, and quality assurance. Another strategy is to use a mini-ridge filter to widen the Bragg peak width in the longitudinal direction for low-energy beams, decreasing the number of energy layers needed [3,4,5,6,7]. However, the high-energy beams do not need a mini-ridge filter to widen the Bragg peak width. It is common to have the synchrotron nozzle to operate in either with or without a mini-ridge filter mode [8]. In addition to the time of taking out and putting in the mini-ridge filter, the disadvantages with this approach are, again, increased burdens for commissioning, TPS beam modeling, and quality assurance, as well as safety concerns in the event of interlock failure. To facilitate clinical use, a mini-ridge filter was designed, which was thick enough to increase the Bragg peak width to create a uniform spread-out Bragg peak (SOBP), yet thin enough to have only a small effect on spot size for the low-energy beams. At the same time, the mini-ridge filter had a minimal effect on the high energies; therefore, it could be kept in the treatment nozzle permanently, which is the concept of an integrated mini-ridge filter (IMRF).

Many questions need to be answered before the IMRF is used clinically. The first is whether treatment plans with an IMRF meet clinical requirements, since the IMRF not only increases the Bragg peak widths but also slightly increases the spot sizes, especially for the low-energy beams. Therefore, the IMRF is expected to slightly increase the lateral and distal penumbras of the SOBP. The second question is whether the IMRF will affect treatment plans with high-energy beams. The third question is whether the efficiency gained using an IMRF through the reduction of energy layers is worthwhile. The aim of this work is to address these questions. We compared the dosimetric characteristics, plan quality, and treatment delivery efficiency between a PBS nozzle with an IMRF and one without an IMRF to understand the feasibility of IMRFs for a synchrotron system.

## 2. Results

### 2.1. Effect of IMRF on Integral Depth Dose and Spot Size

**Figure 1:**
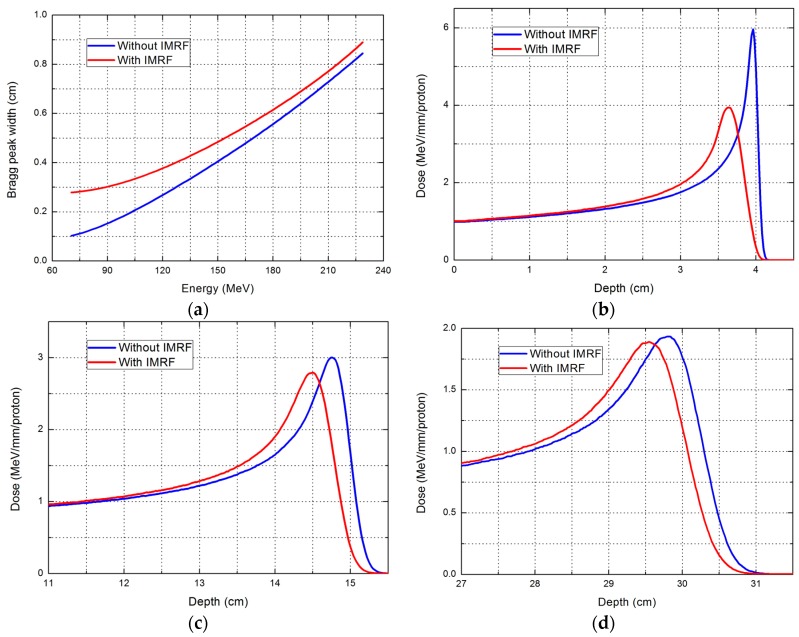
Bragg peak width (defined at 80% the peak) and integral depth dose for plans with and without integrated mini-ridge filter (IMRF). (**a**) Bragg peak width; (**b**) range = 4 cm; (**c**) range = 15 cm; (**d**) range = 30 cm.

With the IMRF, the Bragg peak width (defined as the width between the proximal 80% and the distal 80% dose curve) was increased by 0.18 cm at the lowest energy, about 70 MeV, and the increase was only about 0.04 cm at the highest energy. Examples of pristine Bragg peaks with and without IMRF for low, medium, and high energies are shown in Figure 1.

**Figure 2:**
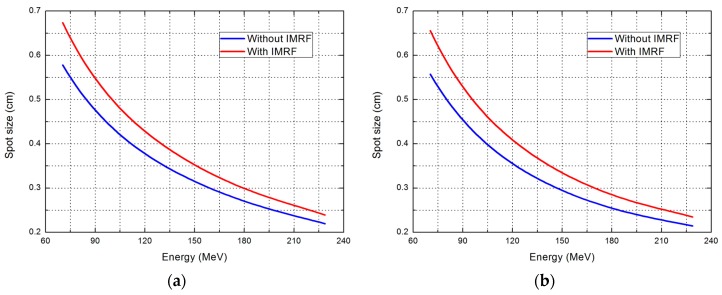
Spot size (1σ) for plans with and without IMRF as a function of energy. (**a**) spot size in cross-line direction; (**b**) spot size in in-line direction.

Figure 2 shows spot size (1σ) for plans with and without IMRF. The spot size increased by about 0.1 cm at the lowest energies, and the increase was only 0.02 cm at the highest energy (about 220 MeV).

### 2.2. Phantom Planning Study

Table 1 lists the number of layers, number of post-processed spots, and beam delivery time for an SOBP of 4 cm with various ranges. The IMRF substantially reduced the number of layers, number of post-processed spots, and beam delivery time for ranges less than 10 cm. However, this reduction diminished as the range increased. These diminishing gains were expected: at low energies, the range intervals were 3 mm with IMRF and 1 to 2 mm without IMRF but became more similar between the plans as the energy increased; thus, the number of layers and beam delivery times also became more similar as energy increased.

The IMRF also increased the lateral and distal penumbras (Table 2). The increase in the lateral penumbra ranged from 0.02 to 0.12 cm, and these increases became smaller as energy increased. The difference in the distal penumbra was negligible, with a maximum value of 0.06 cm.

**Figure 3:**
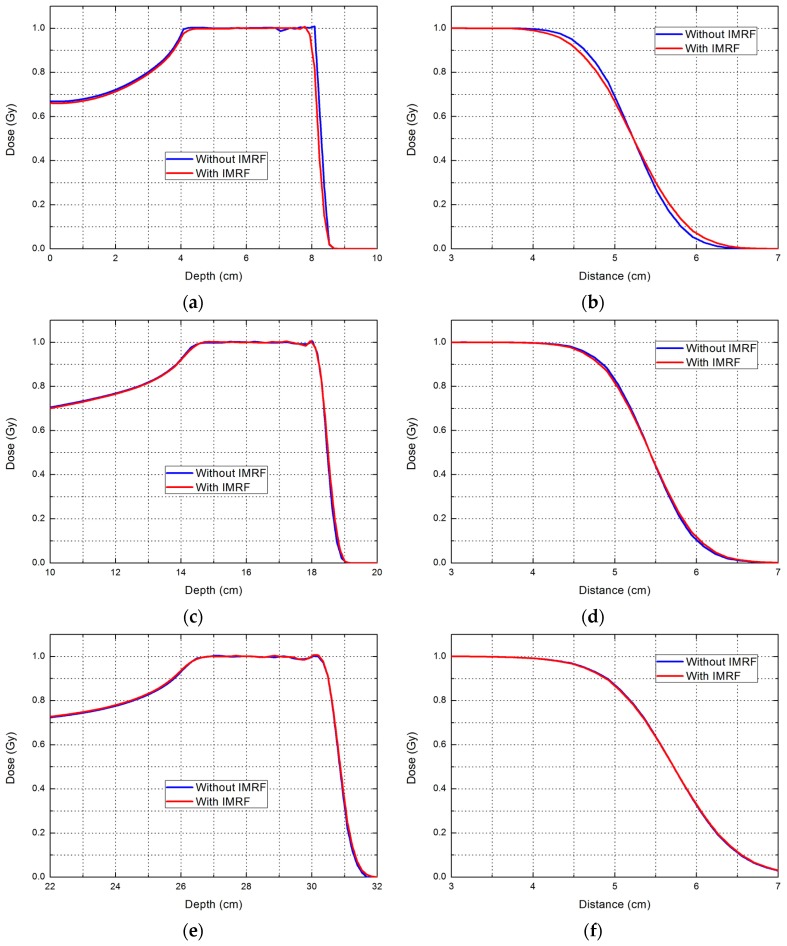
Spread-out Bragg peak (left column) and lateral profile (right column) for three ranges with a spread-out Bragg peak of 4 cm and a field size of 10 × 10 cm^2^. The depth of the lateral profile is selected at the middle of the spread-out Bragg peak. The range is 8 cm for (**a**,**b**); 18 cm for (**c**,**d**); and 30 cm for (**e**,**f**).

The shape of the SOBP, represented by depth dose curves, is the key to the IMRF design. Figure 3 allows comparison of the SOBP and lateral profile for low, medium, and high energies. The SOBP shapes and the lateral profiles at medium and high energies were identical between the plans with and without IMRF. At the low energies, different range intervals (3 mm with IMRF and 1 to 2 mm without IMRF) showed small differences between plans at the distal edge. These increases in range intervals due to IMRF at low energies did not create ripples of SOBP; the plans with IMRF had the same dose uniformity as the plans without IMRF. This finding confirmed that the IMRF was thick enough to create uniform doses with 3 mm range spacing for the low-energy beams.

### 2.3. Clinical Case Planning Study

Table 3 lists the numbers of layers, raw spots, and post-processed spots and beam delivery time for each clinical plan. For the prostate case, which mainly used high energies, the IMRF had no effects on the number of layers and the beam delivery time. For the other cases, which used more low energies, the number of layers was markedly reduced for the plan with IMRF compared with the plan without IMRF; the reductions ranged from 10.3% to 50.6%. For the cases that used low energies (all except the prostate case), the beam delivery time ranged from 188.5 s to 526.3 s without IMRF but ranged from 99.7 s to 392.3 s with IMRF; this result indicates that IMRF achieved beam delivery time reductions of 9.3% to 47.1%.

**Figure 4:**
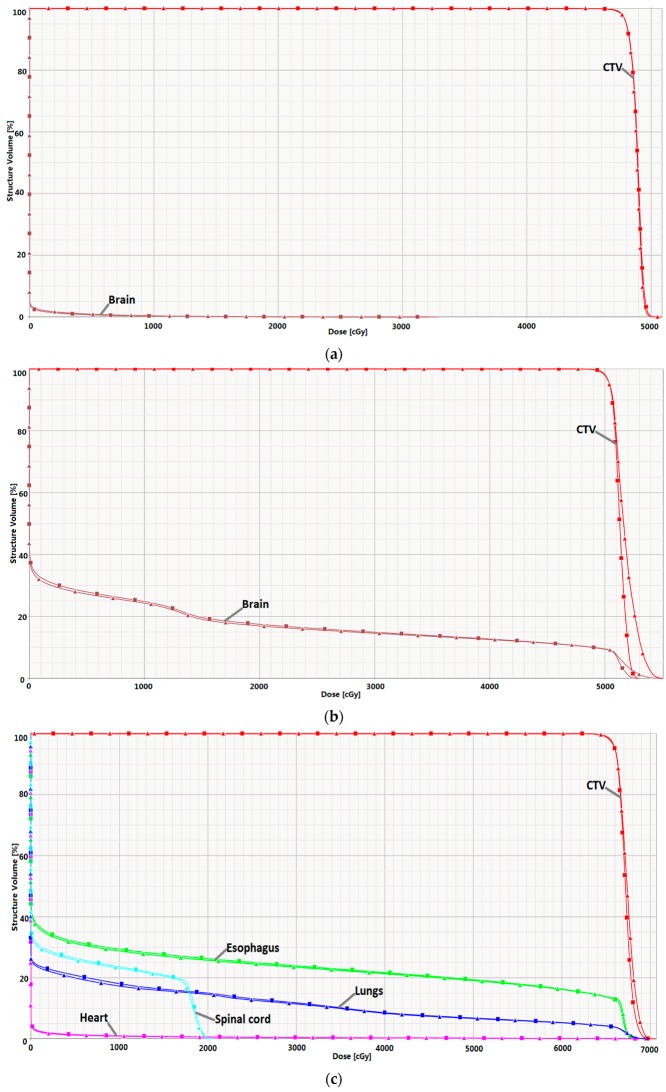

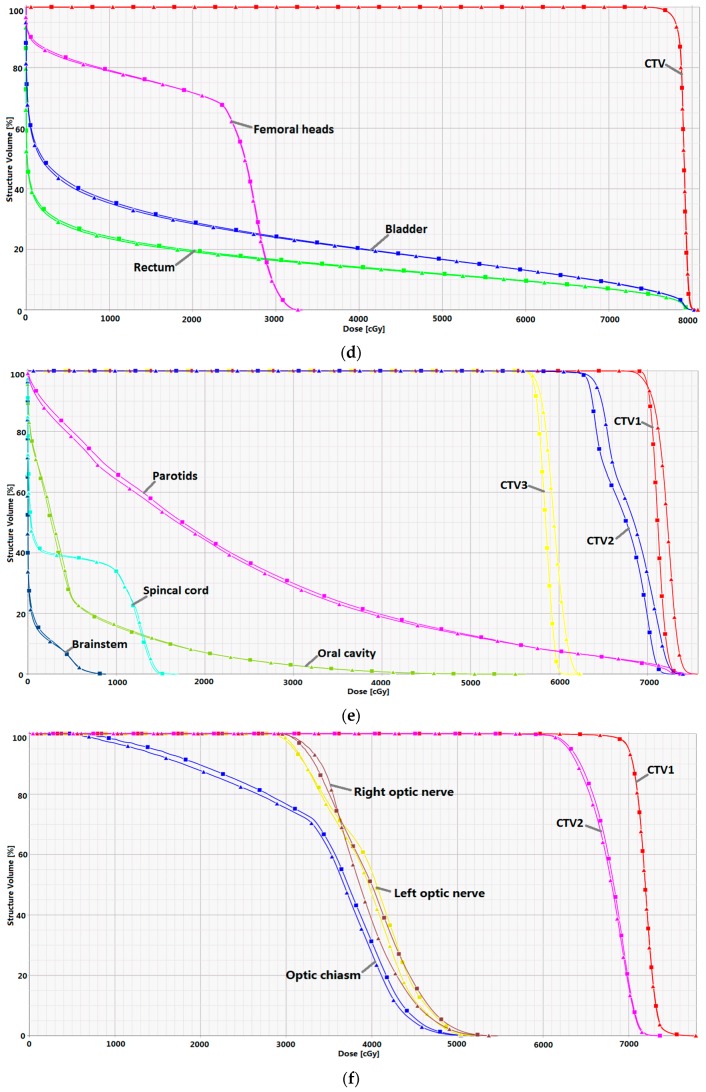
Dose-volume histograms with IMRF (line with squares) and without IMRF (line with triangles). (**a**) scalp case; (**b**) brain case; (**c**) lung case; (**d**) prostate case; (**e**) head and neck (H & N) case; and (**f**) base of skull (BOS) case. CTV, clinical target volume.

Figure 4 allows comparison of the dose–volume histograms for the selected cases. All dosimetric evaluation criteria are listed in Table 4. IMRF had a negligible effect on doses to the brain for the brain case, increasing the brain D_mean_ by 1.1%. For the lung case, IMRF increased the lung V_20Gy_ from 14.5% to 15.0%, did not change the spinal cord D_max_, and increased the esophagus V_60Gy_ by 0.6%. For the prostate case, the dose–volume histograms for the bladder, rectum, and femoral heads were identical between the plans with and without IMRF. For the H & N case, IMRF increased the parotids D_mean_ by 2.7% but reduced the oral cavity D_mean_ by 1.8% (Figure 4e). For the base of skull (BOS) case, the maximum dose D_max_ to the left optic nerve, right optic nerve, and optic chiasm revealed minor differences due to IMRF, with a maximum difference of 1.2%. For all cases selected for this study, radiation oncologists in our center reviewed the plans with and without IMRF and determined that the doses to the organs at risk were clinically equivalent between the plans.

Table 4 lists the HIs (HI, homogeneity index) and CIs (CI, conformity index) for all six cases. The IMRF enhanced the target dose uniformity in five of the cases (excluding the BOS case); this increase due to IMRF ranged from 0.1% to 2.9%. As mentioned, CTV1 was used as the representative target for the H & N and BOS cases. There was no obvious pattern of change in CI across cases; compared with the plans without IMRF, the plans with IMRF had slightly lower CIs for the scalp and BOS cases but larger CIs for the brain and H & N cases. Overall, IMRF had a very small effect on doses in treatment plans since IMRF was accompanied by only a small increase in spot size, even at the low energies.

## 3. Discussion

Our mini-ridge filter design effectively broadened the Bragg peaks in the low-energy beams and had a minimal impact on the high-energy beams. The increase in Bragg peak width due to the IMRF was about 2 mm at the lowest energy. The maximum increase in spot size due to the IMRF was within 1 mm, and the maximum increase in penumbra was also about 1 mm. For the phantom study, the maximum reduction in treatment time due to the IMRF was 53.3% and was seen with the plan with a range of 8 cm and an SOBP width of 4 cm. For the clinical cases, the IMRF reduced the estimated treatment delivery time for plans requiring low and middle energies, with reductions ranging from 9.3% to 47.1%. For the prostate case, which used high and middle energies, beam delivery time did not differ much between plans with and without IMRF (−0.2%). There were only small dosimetric differences between plans. An added benefit of IMRF was that the minimum monitor unit (MU) effect was reduced for the plans using low energies, as demonstrated by the somewhat larger HI values for the H & N and brain cases with IMRF. All of these results suggest that one proton scanning nozzle of Hitachi systems (Hitachi America Ltd., Tarrytown, NY, USA) with an IMRF is feasible, and the idea of IMRF could also hold for other similar systems.

The trend of proton therapy is moving from passive scattering to PBS. The latter allows intensity-modulated proton therapy [9,10]. The sharp Bragg peaks at the low energies from a synchrotron system are a potential concern for treatment delivery efficiency. Two recent studies for miniRF focused on motion mitigation of PBS [3,4]. Courneyea et al. [3] studied various thicknesses of mini-ridge filter in connection with breath-hold treatment delivery for moving target volumes and recommended using the thinnest mini-ridge filter that achieved the desired timing for breath-hold treatments. Matsuura et al. [4] developed a mini-ridge filter for their respiratory-gated PBS delivery; they found that energy layers could be reduced by up to 50% and that target volume coverage parameters were within 1% for shallow target volumes. Our results on energy layer reduction for low-energy beams are consistent with this finding. In the current study, we focused on whether a mini-ridge filter can be permanently placed in the scanning nozzle of the latest generation of Hitachi synchrotron systems. With the IMRF, the advantages of a mini-ridge filter for breath–hold treatments and respiratory gating could be realized as well.

Spot size is an important parameter for PBS. The smaller the spot size, the steeper the dose gradient and the better the normal tissue can be protected [11]. The IMRF increased the spot size. The spot size of the synchrotron system without IMRF is comparable to a commercial cyclotron system at low energies. Owing to the IMRF, the spot size is slightly larger than the cyclotron system for low energies (<90 MeV), but still smaller for higher energies (>100 MeV) [12]. For the clinical cases, the IMRF slightly increased the dose to organs at risk, but a team of radiation oncologists determined that the increase would not have a marked clinical impact.

The IMRF enhanced the target dose uniformity, particularly for the H & N and brain cases. The reason for this enhancement is that the impact of the minimum MU constraint was reduced with the IMRF [13]. The optimization process optimized the weight of each raw spot, but the raw spot was going through a rounding process in post-processing if the weight was less than the minimum MU per spot constraint (0.003 MU). The dose used for optimization iteration and the dose after post-processing could be different if there were a large number of raw spots with weights less than the minimum MU. For example, in the H & N case, the raw spots were removed more than 1/3 in the post-process for the plan without IMRF but were removed less than 1/4 for the plan with IMRF. Therefore, the target dose uniformity of the plan with IMRF was better than that of the plan without IMRF.

Robustness against uncertainties is another important aspect of an IMPT plan. Studies have found that the greater the spot size, the more robust the treatment plan [14]. In this study, we utilized the dose–volume histogram bands method, with 3.5% range uncertainties and 3 mm setup uncertainties, to validate the robustness of the plans with and without IMRF. While no difference was found between the plans, considering the small increase in the spot sizes at the low energies, one could expect that the robustness of plans with IMRF would be equal to or better than that of plans without IMRF.

**Figure 5:**
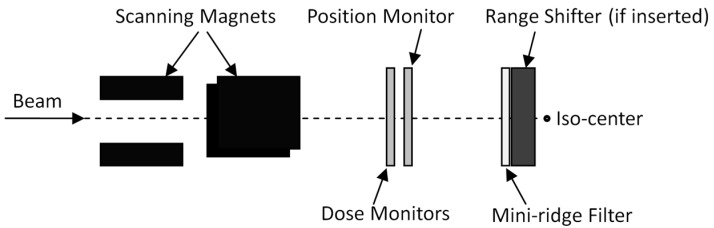
Schematic of the IMRF.

In this study, the beam data required by the TPS were provided by the vendor, so the hardware design details of the IMRF were not discussed. We noticed that the design of the IMRF has many factors to consider [3]. In this study, the mini-ridge filter was placed just above the range shifter, as shown in Figure 5. A possible alternative location could be near the exits of the synchrotron. With this placement, some of the phase space parameters of the proton beam could be recovered by the magnets along the beam transport line so that the spot size would not be affected. The convenient replacement of the mini-ridge filter is another concern. Since the mini-ridge filter is a small structure in the synchrotron nozzle, slight damage could alter its dosimetric characteristics, so the design of the mini-ridge filter should be easy to replace when there is damage or other issues.

A rigorous quality assurance (QA) program for IMRF, including physical integrity of the device and dosimetric characterizes, should be implemented before it is used clinically. Fortunately, the routine dosimetric QA, including spot sizes and beam ranges for different energies, would detect change of the device. It should be emphasized that the dosimetric characteristics should be checked not only along the central axis, but also randomly selected points off-axis in a periodic QA program.

In addition to IMRFs, a possible method of reducing energy layers is energy optimization, presented previously by Cao et al. [15]. On the accelerator side, a new technology allowing the extraction of multiple energies may be available soon [16]. An IMRF could be combined with energy optimization and/or multiple-energy extraction to further reduce the number of energy layers.

## 4. Materials and Methods

### 4.1. Treatment Delivery System

This study was conducted using one of the latest generations of synchrotron systems (Hitachi America Ltd., Tarrytown, NY, USA). The Hitachi synchrotron system has 94 energies ranging from 4.0 to 30.6 g/cm^2^. The range intervals of the beams increase from 0.1 to 0.6 cm as the energy increases. The selection of these intervals is based on creating uniform SOBPs with ripples of less than 3% [8]. To aid discussion in this study, we divided these energies into three groups on the basis of their range in water: low energies (72.5 to 117.3 MeV), middle energies (118.6 to 173.7 MeV), and high energies (176.2 to 221.8 MeV), corresponding to ranges of 4.0 to 9.9 g/cm^2^, 10.1 to 20.0 g/cm^2^, and 20.5–30.6 g/cm^2^, respectively. A 4.0 cm range shifter was used when there was a shallow target (the minimum range was less than 4 g/cm^2^). The minimum and maximum MU values for each spot were 0.003 and 0.04, respectively.

The IMRF was designed to broaden the pristine Bragg peaks such that uniform SOBPs were generated for the lowest energies while keeping the IMRF as thin as possible to minimize its effects on the high energies. For the PBS nozzle with IMRF, the range intervals were 0.3, 0.4, and 0.6 cm as the energy increased. The selection of these intervals was also based on creating uniform SOBPs with ripples less than 3%. Figure 5 is a schematic of the IMRF and its major components: the mini-ridge filter was located near the end of the treatment nozzle just above the range shifter, a mechanism that has been previously described in reference [8]. The data required by the TPS for the IMRF were generated by Monte Carlo simulation and were supplied by the synchrotron system vendor.

### 4.2. Treatment Planning System

The proton beam models using the pencil beam dose algorithm for plans with and without IMRF were commissioned in Eclipse version 13.6 (Varian Medical System, Palo Alto, CA, USA). The grid size for dose calculation was set to 0.2 × 0.2 × 0.2 cm^3^. Considering that the spot spacing and the minimum and maximum MU values of each spot had an impact on plan quality [13,17], we fixed spot spacing at 0.5 cm in this study. The minimum and maximum MU of each spot in the system with IMRF was consistent with those in the system without IMRF (minimum 0.003 MU, maximum 0.04 MU). In treatment planning, all proton doses were expressed in Gy (RBE, relative biological effectiveness) with a constant RBE factor of 1.1. Inverse planning using single-field optimization or multiple-field optimization [13] with simultaneous spot optimization were used for the clinical planning study (described further below). Each spot’s position, energy, and number of MUs were determined using the TPS.

### 4.3. Phantom Planning Study

We evaluated the effect of the IMRF on dosimetric characteristics in a 36 × 36 × 36 cm^3^ homogeneous water phantom. Single-beam plans assuming a fraction dose of 1 Gy were generated to create a uniform depth dose with an SOBP width of 4 cm and a field size of 10 × 10 cm^2^. For a given energy, the weights of all spots were equal. Individual ranges 8, 10, 14, 18, 22, 26, and 30 g/cm^2^ were used to evaluate the effect of the IMRF on lateral and distal penumbras. We defined the lateral penumbra as 80% and 20% dose distance of the lateral profiles and the distal penumbra as the width between the distal 80% and 20% dose curves.

### 4.4. Clinical Case Planning Study

**Figure 6:**
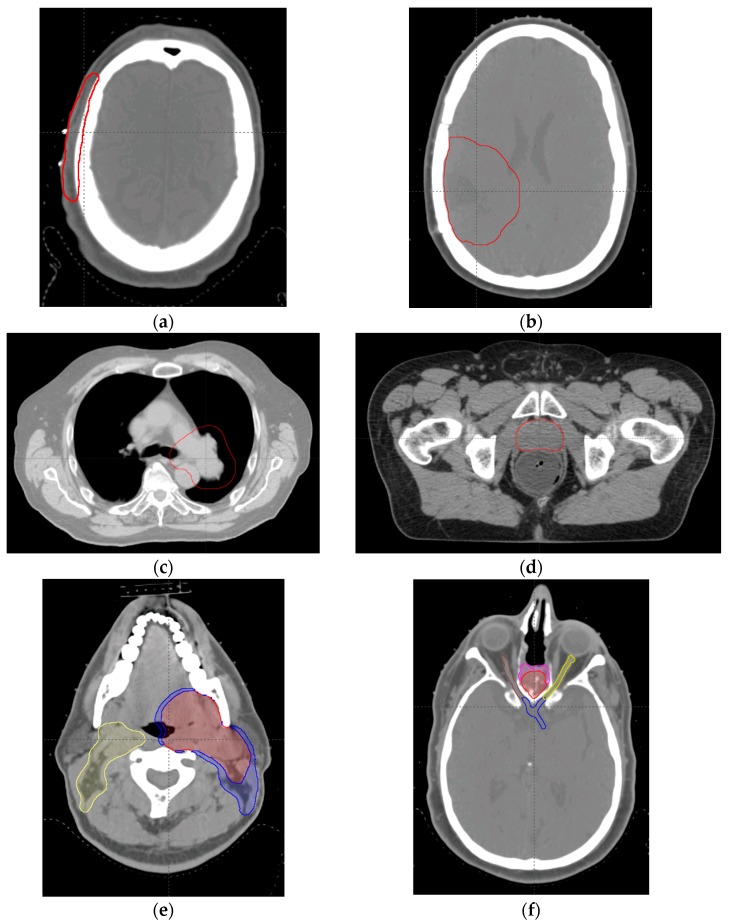
Representative target volumes in the transverse plains for the cases in this study. (**a**) scalp case; (**b**) brain case; (**c**) lung case; (**d**) prostate case; (**e**) H & N case; and (**f**) BOS case. For (**a**–**d**), the red contour is the clinical target volume (CTV). For (**e**), the red, blue, and yellow contours are CTV1, CTV2, and CTV3, respectively. For (**f**), the red and magenta contours are CTV1 and CTV2, respectively; and the yellow, brown, and blue contours are the left optic nerve, right optic nerve, and optic chiasm, respectively.

We then evaluated the effect of the IMRF on plan quality using six clinical cases previously treated at our center with targets in the scalp, brain, lung, prostate, head and neck (H & N), and base of skull (BOS) (one case for each location). Figure 6 shows representative axial images with target volumes for these cases. The scalp case had a shallow target volume and used only the low energies; the brain case had a slightly deeper target volume and used the low and middle energies; the lung case had mean nominal range and nominal SOBP of beams of 18.0 cm and 11.4 cm, respectively, and used mainly the middle energies; the prostate case used the middle and high energies; the H & N case had three target volumes and three beams, had a mean nominal range of 22.7 cm, and used energies from low to high. The BOS case also used energies from low to high, but the targets were surrounded by optic nerves and the optic chiasm; we used this case to demonstrate the effects of enlarged spot size on dose.

Table 5 summarizes the plans for the six cases. The goal of optimization was to make the prescription dose cover the target while minimizing the dose to the organs at risk. The scalp, brain, and prostate cases used single-field optimization; and the lung, H & N, and BOS cases used multiple-field optimization. For each case, a plan with IMRF and a plan without IMRF were optimized using the same objectives and optimization methods. All plans were normalized to ensure that the prescription dose covered 95% of the target volume. For the cases with more than one target volume, the target volume receiving the highest prescription dose (CTV1) was normalized to make the prescription dose cover 95% of the volume.

The dosimetric characteristics of treatment plans with and without IMRF were compared using dose–volume histograms. Clinical evaluation criteria were used to compare the doses to the organs at risk between the two plans. We also used dose homogeneity index (HI) and conformity index (CI) to compare target dose distributions. HI was defined as 100% × (1 − (D_5%_ − D_95%_)/D_pre_), in which D_x%_ is the minimum dose to x% of the volume and D_pre_ represents the prescription dose. The larger the HI (maximum 100%), the more uniform the dose in the target area. CI was defined as 100% × (V_(t,ref)_/V_t_) × (V_(t,ref)_/V_ref_), in which V_(t,ref)_ is the target volume covered by the reference isodose, V_t_ represents the target volume, and V_ref_ is the volume covered by the reference isodose. The larger the CI (maximum 100%), the more the dose conforms to the target. Considering that the H & N and BOS cases had multiple targets, we used the target prescribed the highest dose (CTV1) as the representative target volume for HI and CI analysis.

### 4.5. Beam Delivery Efficiency

For both the phantom and the clinical case studies, the number of energy layers, total number of post-processed spots, and beam delivery times were compared between the plans with and without IMRF. The total number of raw spots was compared between the plans with and without IMRF for the clinical case study only. These measures together represented the treatment efficiency of the systems with and without IMRF. Beam delivery time was calculated by a method described previously [18]. We analyzed three categories of delivery time: layer switch time, spot spill time, and spot switch time. Different energies have different layer switch times, effective scanning magnet speeds, and proton spill rates, but these differences are minor. To simplify calculation, we assumed that, for all energies, the layer switch time was 1.9 s; the effective scanning magnet speeds for *x*- and *y*-directions were 6 m/s and 20 m/s, respectively; the proton spill rate was 9 MU/s; and the magnet preparation and verification time was 1.93 × 10^−3^ s [18].

## 5. Conclusions

Integrating a mini-ridge filter into a latest generation of Hitachi synchrotron scanning nozzle is feasible for improving treatment efficiency without significant sacrifice to the plan quality. In some cases, such as brain and H & N plans that use low energies, the dose uniformity can be slightly improved with the IMRF owing to the reduced minimum MU effect. A study with different disease sites and a large number of cases for each site will be required to confirm these findings.

## Figures and Tables

**Table 1:** Beam line information and beam delivery time for the phantom. The spread-out Bragg peak width is 4 cm for all ranges.

Range (cm)	Parameter	Without IMRF	With IMRF	Reduction (%)
8	Number of layers	36	14	61.1%
	Post-processed spots	10,693	4624	56.8%
	Beam delivery time (s)	100.3	46.8	53.3%
10	Number of layers	24	14	41.7%
	Post-processed spots	7225	4624	36.0%
	Beam delivery time (s)	70.1	46.1	34.2%
14	Number of layers	16	15	6.3%
	Post-processed spots	5202	4913	5.6%
	Beam delivery time (s)	50.2	47.9	4.6%
18	Number of layers	11	11	0.0%
	Post-processed spots	3757	3757	0.0%
	Beam delivery time (s)	37.4	37.5	−0.3%
22	Number of layers	9	9	0.0%
	Post-processed spots	3179	3179	0.0%
	Beam delivery time (s)	32.5	32.6	−0.3%
26	Number of layers	8	8	0.0%
	Post-processed spots	2890	2890	0.0%
	Beam delivery time (s)	29.7	30.0	−1.0%
30	Number of layers	8	8	0.0%
	Post-processed spots	2890	2890	0.0%
	Beam delivery time (s)	29.8	29.8	0.0%

**Table 2:** Lateral and distal penumbras for the phantom. The spread-out Bragg peak width is 4 cm for all ranges.

Range (cm)	Lateral Penumbra (cm)		Distal Penumbra (cm)	
	Without IMRF	With IMRF	Without IMRF	With IMRF
8	0.77	0.89	0.26	0.26
10	0.73	0.83	0.25	0.28
14	0.71	0.78	0.30	0.34
18	0.75	0.80	0.35	0.39
22	0.83	0.87	0.42	0.44
26	0.93	0.96	0.44	0.50
30	1.05	1.07	0.51	0.54

**Table 3:** Beam lines and beam delivery times for plans without and with integrated mini-ridge filter (IMRF).

Case	Parameters	Without IMRF	With IMRF	Reduction
Scalp (2 beams)	Number of layers	50 + 33 = 83	27 + 14 = 41	50.6%
	Raw spots	5681 + 5268 = 10,949	2227 + 2089 = 4316	60.6%
	Post-processed spots	5417 + 4654 = 10,071	2622 + 2461 = 5083	49.5%
	Beam delivery time (s)	111.2 + 77.3 = 188.5	62.4 + 37.3 = 99.7	47.1%
Brain (3 beams)	Number of layers	36 + 39 + 30 = 105	27 + 23 + 27 = 77	26.7%
	Raw spots	3831 + 5442 + 2830 = 12,103	3038 + 2937 + 2733 = 8708	28.1%
	Post-processed spots	2947 + 3385 + 2174 = 8506	2665 + 2498 + 2148 = 7311	14.0%
	Beam delivery time (s)	76.5 + 83.2 + 63.1 = 222.8	58.9 + 51.2 + 57.4 = 167.5	24.8%
Lung (3 beams)	Number of layers	43 + 39 + 50 = 132	38 + 31 + 35 = 104	21.2%
	Raw spots	7182 + 7991 + 8188 = 23,361	6244 + 6363 + 6351 = 18,958	18.8%
	Post-processed spots	6440 + 6740 + 7023 = 20,203	5866 + 5978 + 6249 = 18,093	10.4%
	Beam delivery time (s)	100.4 + 92.8 + 114.7 = 307.9	89.6 + 76.3 + 84.9 = 250.8	18.5%
Prostate (2 beams)	Number of layers	19 + 18 = 37	19 + 18 = 37	0.0%
	Raw spots	1686 + 1639 = 3325	1767 + 1694 = 3461	−4.1%
	Post-processed spots	1566 + 1536 = 3102	1635 + 1595 = 3230	−4.1%
	Beam delivery time (s)	41.8 + 39.8 = 81.6	41.9 + 39.9 = 81.8	−0.2%
H & N (3 beams)	Number of layers	74 + 71 + 71 = 216	48 + 52 + 53 = 153	29.2%
	Raw spots	19,688 + 19,506 + 22,670 = 61,864	14,144 + 14,678 + 15,230 = 44,052	28.8%
	Post-processed spots	13,193 + 12,560 + 15,342 = 41,095	11,426 + 9960 + 11995 = 33,381	18.8%
	Beam delivery time (s)	177.8 + 170.0 + 178.5 = 526.3	125.5 + 129.0 + 137.8 = 392.3	25.5%
BOS (3 beams)	Number of layers	46 + 41 + 39 = 126	41 + 37 + 35 = 113	10.3%
	Raw spots	4878 + 6171 + 6443 = 17,492	4735 + 5755 + 5897 = 16,387	6.3%
	Post-processed spots	3086 + 4026 + 4165 = 11,277	2935 + 3915 + 4073 = 10,923	3.1%
	Beam delivery time (s)	96.2 + 89.6 + 86.0 = 271.8	86.5 + 81.8 + 78.3 = 246.6	9.3%

Abbreviations: H & N, head and neck; BOS, base of skull.

**Table 4:** Dosimetric comparison of plans without and with IMRF.

Case	Parameters	Without IMRF	With IMRF
Scalp	HI	96.8	97.0
	CI	84.5	81.1
	Brain D_mean_ (Gy)	0.1	0.1
Brain	HI	93.7	96.4
	CI	92.6	93.7
	Brain D_mean_ (Gy)	9.0	9.1
Lung	HI	95.6	96.2
	CI	93.0	93.0
	Lungs V_20Gy_ (%)	14.5	15
	Spinal cord D_max_ (Gy)	20.5	20.5
	Esophagus V_60Gy_ (%)	16.2	16.3
Prostate	HI	97.8	97.9
	CI	91.9	92.4
	Bladder V_70Gy_	9.2	9.0
	Rectum V_70Gy_	6.9	7.0
	Femoral heads D_mean_ (Gy)	21.0	21.1
H & N	HI	94.7	96.5
	CI	76.1	83.6
	Oral cavity D_mean_ (Gy)	5.5	5.4
	Spinal cord D_max_ (Gy)	17.0	16.7
	Brainstem D_max_ (Gy)	8.8	8.9
	Parotids D_mean_ (Gy)	22.4	23.0
BOS	HI	95.0	94.9
	CI	85.3	84.4
	Left optic nerve D_max_ (Gy)	52.1	52.6
	Right optic nerve D_max_ (Gy)	54.6	54.8
	Optic chiasm D_max_ (Gy)	52.1	52.7

Abbreviations: HI, homogeneity index; CI, conformity index.

**Table 5:** Treatment planning information for the cases selected for this study.

Case	Target Volume (cc)	Number of Beams	Optimization Method	Prescription (Gy (RBE)/Fraction)
Scalp	CTV, 47.3	2	SFO	48/24
Brain	CTV, 132.7	3	SFO	50.4/28
Lung	CTV, 582	3	MFO	66/33
Prostate	CTV, 88.8	2	SFO	78/39
H & N	CTV1, 161.0	3	MFO	70/30
	CTV2, 83.5			63/30
	CTV3, 225.1			57/30
BOS	CTV1, 142.0	3	MFO	70/30
	CTV2, 42.5			63/30

Abbreviations: cc, cubic centimeter; RBE, relative biological effectiveness; CTV, clinical target volume; SFO, single-field optimization; MFO, multiple-field optimization.
